# Chitosan Nanoparticles Loaded with *Capparis cartilaginea* Decne Extract: Insights into Characterization and Antigenotoxicity In Vivo

**DOI:** 10.3390/pharmaceutics15112551

**Published:** 2023-10-29

**Authors:** Asmaa S. Salman, Shaza N. Alkhatib, Fatimah M. Ahmed, Ragaa A. Hamouda

**Affiliations:** 1 Biology Department, College of Science and Arts at Khulis, University of Jeddah, Jeddah 21959, Saudi Arabia; drasmaasalman@gmail.com (A.S.S.); snalkhatib@uj.edu.sa (S.N.A.); mothanna.fatima@gmail.com (F.M.A.); 2 Genetic and Cytology Department, Biotechnology Research Institute, National Research Center, Cairo 12622, Egypt; 3 Microbial Biotechnology Department, Genetic Engineering and Biotechnology Research Institute (GEBRI), University of Sadat City, Sadat City 32897, Egypt

**Keywords:** chitosan, nanoparticles, *Capparis cartilaginea*, genotoxicity, sperm

## Abstract

Plant-based foods may enhance the prevention of cancer. The present investigation aimed to assess the antigenotoxic effects of chitosan nanoparticles (CNPs) when loaded with the ethanol extract of *C. cartilaginea* (CNPs/Cc). Synthesis of CNPs and CNPs/Cc and their characterization were carried out using TEM, EDS, DSC, and Zeta potential. For in vivo experiments, animal groups were treated in the following groups: negative control, ethyl methanesulfonate (EMS) (240 mg/kg), CNPs (350 mg/kg), high and low doses of CNPs/Cc, CNPs plus EMS, high dose of CNPs/Cc plus EMS, and low dose of CNPs/Cc plus EMS. Bone marrow chromosomal aberrations and sperm shape abnormalities were examined. TEM results showed that CNPs and CNPs/Cc are spherical particles. CNPs’ physical stability was observed to be lower than that of CNPs/Cc due to the presence of more positive charges on CNPs/Cc. EMS significantly enhanced chromosomal abnormalities and sperm shape abnormalities. CNPs showed powerful antigenotoxic properties. For the first time, it could be concluded that loading chitosan nanoparticles with *C. cartilaginea* extract significantly promotes its protective properties.

## 1. Introduction

Cancer is the second leading cause of death worldwide [1,2]. Environmental and dietary chemicals can induce endogenous and exogenous DNA damage, which is the first step in the process of carcinogenesis [3]. Given the relationship between food, nutrition, and cancer, plant-based diets have been reported to decrease overall cancer risk [4]. 

Medicinal plants with preventive and therapeutic properties play a vital role in healthcare systems. *Capparaceae* comprises a medium-sized family of about 45 genera and 700–900 species, whose members exhibit considerable variation in their habitat [5]. *Capparis* is the largest genus of *Capparidaceae*, comprising about 250–400 species of shrubs, trees, and woody climbers [6], represented by four species in Saudi Arabia. *Capparis C. cartilaginea* Decne is used in traditional medicine for treatment of various illnesses. These plants have shown different biological effects, including antioxidant, antihyperglycemic, hypolipidemic, and analgesic effects [7,8]. There is a growing body of literature that recognizes the immunostimulant, antitumoral, antidiabetic, antisclerotic, antimicrobial, anti-inflammatory, immunomodulatory, and antiviral activities of the *Capparis* species [9].

Currently, the development of new therapeutic drugs with improved efficiency is gaining much interest in the field of plant-based medicines. Nanoscale systems for drug delivery reduce the drug particles into the sub-micron range, thus enhancing the solubility, permeability, absorption, and bioavailability of their active ingredients [10]. One such system uses nanoparticles formed from chitosan, which is the second most abundant natural polymer after cellulose. Chitosan is a nontoxic, biocompatible, biodegradable, and cationic polysaccharide, which can be easily crosslinked with tripolyphosphate (TPP polyanions) under mild conditions to form nanoparticles [11,12]. The use of chitosan nanoparticles, with a tunable size and the possibility of surface modification, is a very promising and versatile strategy to overcome the bioavailability and stability issues of diverse natural active ingredients [13]. Chitosan-based nanosystems are among the most important and advanced drug delivery systems due to their remarkable physicochemical and biological characteristics. Thus, it has been widely used in the medical and pharmaceutical fields, and its potential as a drug carrier has been considered [14]. 

Ethyl methanesulfonate (EMS) is a dangerous chemical substance: it is mutagenic, teratogenic, and carcinogenic, and neither occurs naturally nor has any commercial uses. However, EMS is a known trace impurity in medications made of mesylate salts. Thus, it is regarded as an impurity rather than a contamination. EMS can cause gene mutations and chromosomal abnormalities through nucleotide substitution, especially through G:C-to-A:T transitions brought on by guanine alkylation [15,16,17]. In biochemical and medical research, EMS is generally utilized as a model alkylating agent, particularly in studies of DNA repair mechanisms. Usually, the mutations are severe loss-of-function or null alleles. Deletions or other chromosomal rearrangements account for around 13% of the EMS lesions [18].

Multiple phenotypes, metabolic byproducts, and biotic/abiotic stress tolerance are displayed in the EMS-induced mutants [19]. Guruprasad [20] observed that *Brahmarasayana* significantly reduced Ethylmethanesulfonate induced chromosomal aberrations in mouse bone marrow cells. The genotoxicity of EMS in mammals like mouse and rats was accomplished with changes in sperm morphology and limb defects in the embryos of pregnant rats [21].

Therefore, the present study aims to synthesize and characterize chitosan nanoparticles loaded with *C. cartilaginea* leaf extract and to establish whether loading chitosan nanoparticles with *C. cartilaginea* extract improves antigenotoxic activity against ethyl methanesulfonate (EMS)-induced genotoxicity in mice.

## 2. Materials and Methods

### 2.1. Materials

The leaves of *C. cartilaginea* were gathered in December 2021 from Al-Hada, Saudi Arabia, and were identified according to Chaudhary [22]. The chemicals used in this study were of analytical grade and used without further purification. Chemicals were purchased from the Saudi Chemical Company (PanReac AppliChem, Ar Riyad, Saudi Arabia).

#### 2.1.1. Preparation of *C. cartilaginea* Leaf Extract

A total of 1031.0 g of *C. cartilaginea* leaves were cleaned and air-dried in the shade, then ground using a suitable grinder. The powdered leaves were soaked in absolute ethanol for 72 h. The extract was filtered, then evaporated using a rotary evaporator (IKA RV 8 Basic V-C, Leicestershire, UK). The leaf extract was lyophilized using a freeze-drying lyophilizer until complete dryness was achieved and preserved at −20 °C. The residue yield was 8.2% of *C. cartilaginea*.

#### 2.1.2. *C. cartilaginea* Characterization

The characterization of *C. cartilaginea* was performed via Fourier-transform infrared spectroscopy (FTIR) using a Thermo Fisher Nicolet IS10 (Waltham, MA, USA) spectrometer.

##### GC–MS Analysis

The method of GC–MS analysis was used on the plant according to Rautela et al. [23].

### 2.2. Synthesis and Optimization Process of CNPs/Cc

#### 2.2.1. Synthesis of CNPs

**Figure 1:**
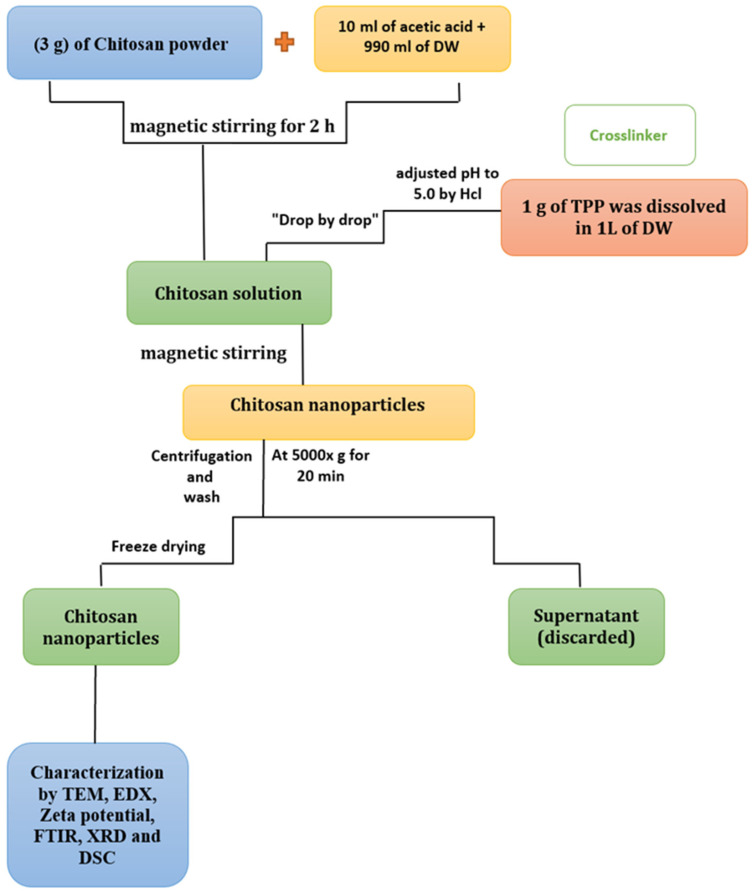
Schematic representation of the CNP synthesis process.

Chitosan was obtained from Sigma-Aldrich. The ionic gelation of CNPs was conducted as described by Masarudin et al. [24]. Three grams of chitosan (medium molecular weight) was mixed with 10 mL of 1.0% acetic acid, and then 990 mL of distilled water was added to the mixture. The solution was stirred using a magnetic stirrer for 2 h to dissolve the chitosan. For the cross-linker, 1 g of TPP was dissolved in 1 L of distilled water at pH 5.0. This was added to the chitosan solution and stirred continuously with a magnetic stirrer until complete dissolution. The mixture was centrifuged at 5000× *g* for 20 min, washed, and then lyophilized until complete dryness (Figure 1).

#### 2.2.2. Synthesis of CNPs/Cc

**Figure 2:**
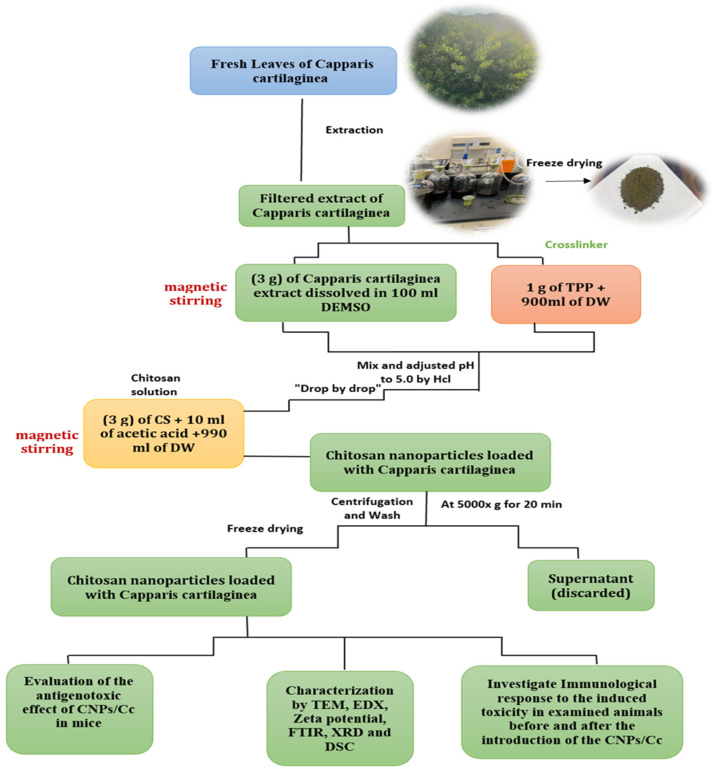
Schematic representation of the CNP/Cc synthesis process.

CNPs and CNPs/Cc were synthesized according to Masarudin et al. [24]. Three grams of chitosan (medium molecular weight) was dissolved in 10 mL of 1.0% acetic acid, then 990 mL of distilled water was added. The chitosan solution was stirred using a magnetic stirrer for two hours to guarantee complete dissolution. The plant extract (3 g) was dissolved in 100 mL of dimethyl sulfoxide (DMSO, purity ≥ 99.5%), using a magnetic stirrer to ensure that the plant extract was completely dissolved. For the cross-linker, 1 g of TPP was dissolved in 900 mL of distilled water at pH 5.0. TPP solution and plant extract were mixed and added drop-by-drop to the dissolved chitosan, then stirred for two hours. The mixture was refined by centrifugation for 20 min at 5000 rpm, then washed three times with distilled water, followed by lyophilization until it was completely dry (Figure 2). This extract was used for pharmacological testing.

#### 2.2.3. Characterization of CNPs and CNPs/Cc

Characterization of CNPs and CNPs/Cc was accomplished using an FTIR (Waltham, MA, USA) Spectrometer, Thermo Fisher Nicolet IS10, to produce the FTIR spectrum (4000 and 400 cm^−1^). The size and morphology of the nanoparticles at the nanoscale were characterized by means of transmission electron microscopy (TEM) (JEOL JSM-6510/v, Tokyo, Japan). The chemical contents of nanoparticles were determined via energy-dispersive spectroscopy (EDS) (JEOL JSM-6510/v, Tokyo, Japan), and a Zeta potential analyzer was used to determine the surface charge and stability [25] (Malvern Zeta size Nano-Zs90, Malvern, PA, USA). Thermographic analysis was performed via differential scanning calorimetry (DSC) testing of 5–10 mg using a DSC131 EVO (Lyon, France).

### 2.3. In Vivo Antigenotoxicity

#### 2.3.1. Experimental Animals

Male SWR mice (9–12 weeks old, 25–27 g each) were obtained from the Animal House Colony at King Fahad Medical Research Centre. Animals were kept in plastic cages under normal conditions of a night–day cycle (12/12 h). Food and water were supplied ad libitum.

Animals were kept in artificially illuminated (12 h dark/light cycle) and thermally controlled (25 ± 1 °C) conditions. Humane care was applied to all animal groups in compliance with the guidelines of the Animal Care and Use Committee of the University of Jeddah (Approval # UJ-21-DR-41).

#### 2.3.2. Experimental Design

Animals were divided into the groups listed in Table 1.

#### 2.3.3. Chromosome Abnormalities Assay

Chromosomal aberration was assessed as described by Moore et al. [26]. For this, animals were injected i.p. with colchicine, 2 h before sacrifice. The method of Yosida and Amano [27] was applied in bone marrow chromosome preparations. One hundred metaphases were examined per animal, and the metaphases with aberrations such as gaps, chromosome or chromatid breakage, and fragments were recognized.

#### 2.3.4. Sperm Morphology Assay

Rasgele’s sperm morphology testing was carried out [28]. Cauda epididymides were crushed in an isotonic sodium citrate solution. Sperms were smeared on slides, fixed, and stained with Eosin Y. In total, 1000 sperms were examined per animal. The head and tail abnormalities of sperm were detected. 

### 2.4. Statistical Analysis

All data are provided as mean values with standard deviations (SDs) based on five replicates and probability values (*p* ≤ 0.05). Tukey’s method and one-way ANOVA (version 16) were used to evaluate the significance of data. 

## 3. Results and Discussion

### 3.1. Fourier-Transform Infrared Spectroscopy (FTIR)

**Figure 3:**
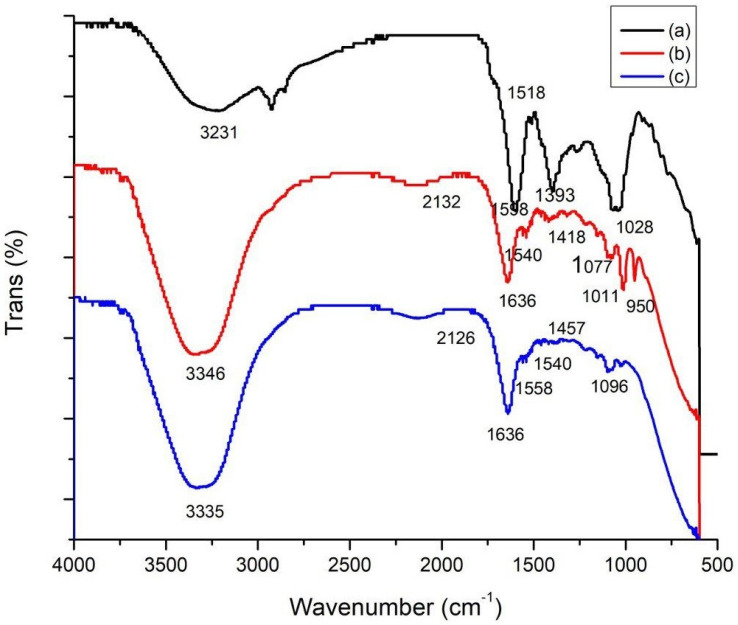
FTIR spectroscopy of *C. cartilaginea* (a), CNPs (b), and CNPs/Cc (c).

FTIR was performed to compare the functional groups of the *C. cartilaginea* extract, the CNPs, and the CNPs/Cc. In total, eight band regions were observed in *C. cartilaginea* (3231, 2924, 1598, 1508, 1396, 1267, 1038, and 614), in the CNPs (3335, 2126, 1636, 1558, 1540, 1457, 1096, and 623 cm^−1^), and in the CNPs/Cc (3346, 2132, 1636, 1540, 1418, 1077, 1011, and 950). The results showed similarity between the chemical structures of CNPs and CNPs/Cc; the wavenumber 1636 was the peak in the CNPs/Cc, and the CNPs presented CHO stretching of the carbonyl group, typical saccharide absorption, and a peak of 1540 cm^−1^, which relates to the amide groups that are present in chitosan (Table 2 and Figure 3).

### 3.2. GC–MS Analysis of C. cartilaginea Extract

**Figure 4:**
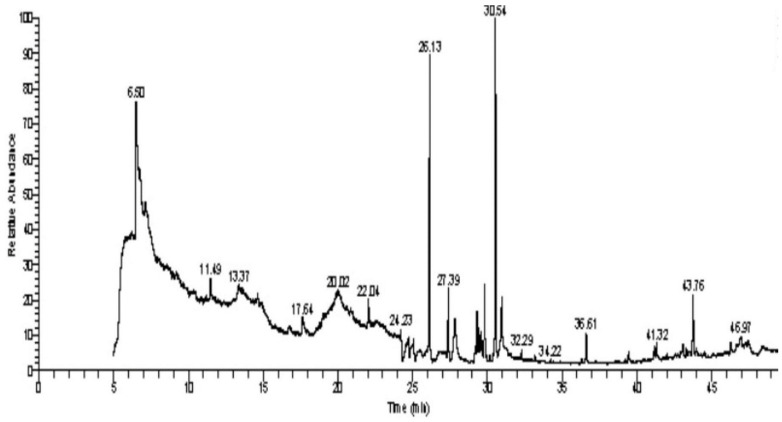
GC–MS analysis of ethanol extracts of *C. cartilaginea* plant.

The GC–MS analysis of *Capparis cartilaginea* showed the presence of 15 compounds (Figure 4). The prevailing compounds were 9,12,15-octadecatrienoic acid, methyl ester (14.99%); Hexadecanoic acid, methyl ester (11.79%); D-Pyroglutamic acid (9.03), hydroxy-bicyclo[3.3.1]n on-2-en-9-one (6.15%); 7-hydroxy-bicyclo[3.3.1]n on-2-en-9-one (4.25%); octadecanoic acid, methyl ester (3.24%); CIS-5,8,11,14,17-eicosapentaenoic acid (3.22%); 9-octadecenoic acid (z)- hexadecanoic ACID (3.21%); hexadecanoic acid, ethyl ester (3.09%); 2-aminoethanethiol hydrogen sulfate (ester) (2.98%); 9-octadecenoic ACID (Z)- 2-aminoethanethiol (2.91%); 9,12-Oetadecadienoyl chloride, (Z,Z)- (2.76%); 9-Octadecenoic acid (Z)-, methyl ester (2.09%); acethydrazide, n2-[1-(2,3-dihydro-6-methyl pyran-2-yl)ethylideno]- (2.05%); 3,5-Heptadienal and 2-ethylidene-6-methyl (2.00%). All these compounds are bioactive and possess many activities such as antibacterial, anticancer, antiviral, and antioxidant, as presented in Table 3. 

### 3.3. Zeta Potential Characterization

**Figure 5:**
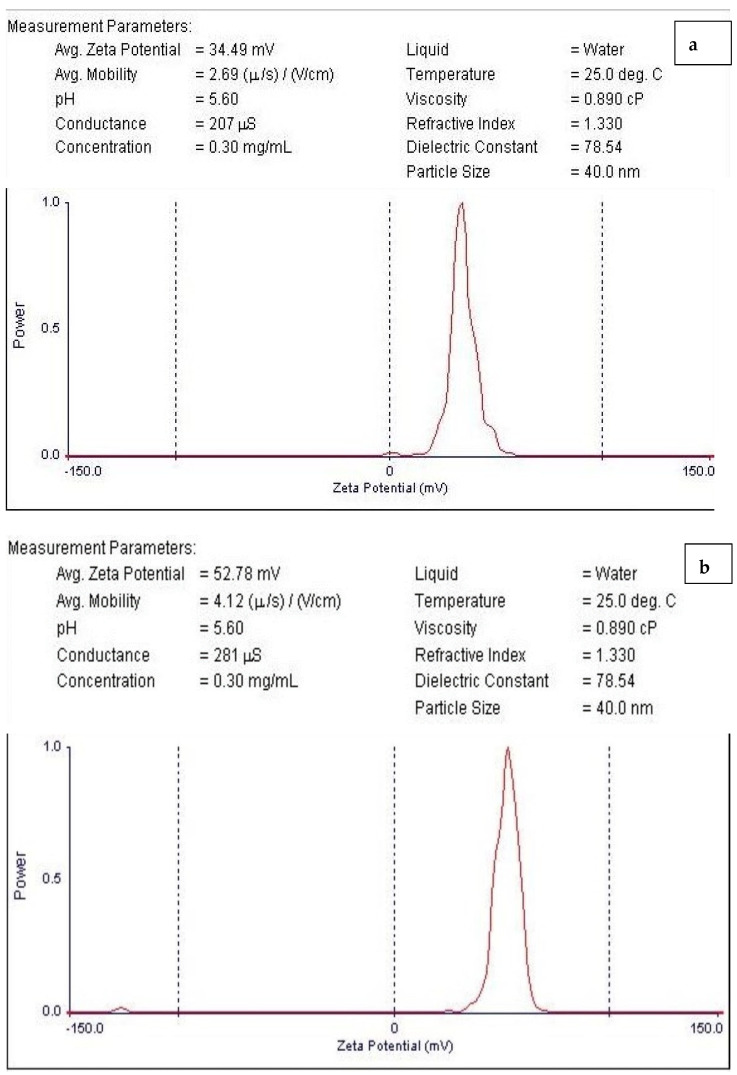
Zeta potential of CNPs/Cc (**a**) and CNPs (**b**).

Zeta potential determination is a crucial method for characterizing the surface charge and comprehending the physical stability of nanosuspensions [50]. The nanoparticles examined in this investigation had surfaces that were positively charged (Figure 5a,b). The results showed that the particles generated in this investigation were comparable to those described in the literature [51,52,53,54,55,56]. The CNPs/Cc had a zeta potential of about +34.49 mV (Figure 5a), while the CNPs had a zeta potential of about +52.78 mV (Figure 5b). This showed the CNPs/Cc’s incipient instability and the CNPs’ moderate stability. The positive surface charges of chitosan were affected by the addition of bioactive components, altering the stability of the nanoparticles. If the value of the zeta potential ranges between 0 and ±5 mV, it is an indicator of rapid coagulation; ±10 to ±30 mV indicates incipient instability; ±30 to ±40 mV indicates moderate stability; ±40 to ±60 mV indicates good stability; and >±61 mV indicates excellent stability [57].

### 3.4. TEM Analysis

**Figure 6:**
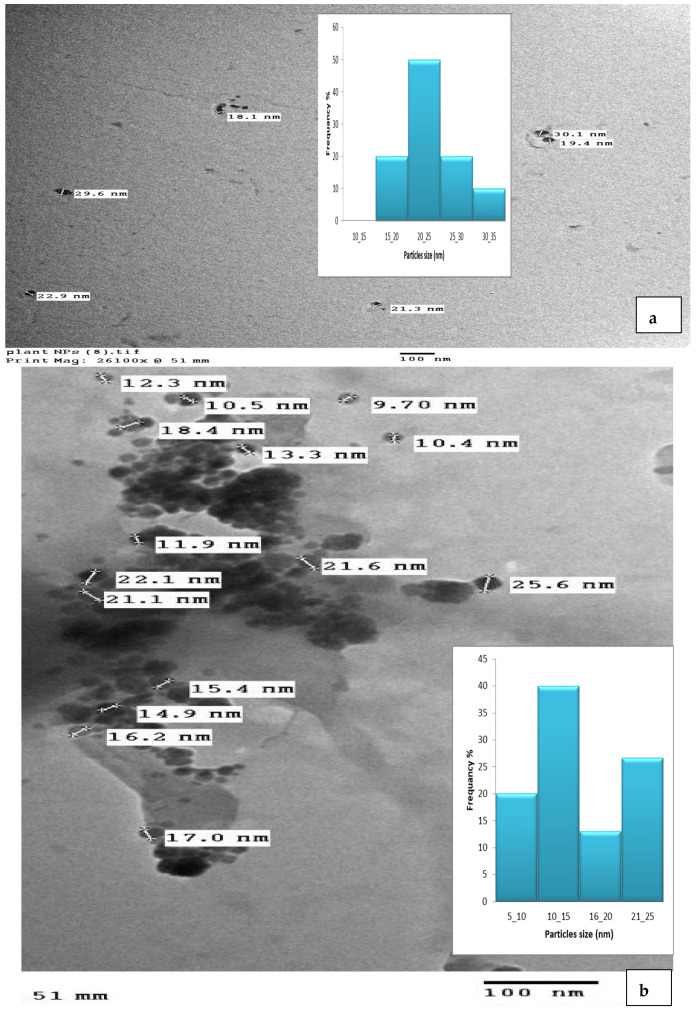
TEM image of CNPs (**a**) and CNPs/Cc (**b**).

A TEM micrograph showed the CNPs to have a spherical particle shape (Figure 6a) and a size range of 9–25 nm [54,56,58], the CNPs/Cc had a spherical shape and a size range of 18–30.1 nm (Figure 6b). The size difference between the CNPs and the CNPs/Cc may be due to the effect of the plant extract being loaded with nanoparticles. Using cross-linked chitosan is necessary to have better control over its shape and size [59]. The TEM image showed the aggregation of the chitosan nanoparticles [60]. The chitosan nanoparticles appeared as tiny and individual spheres, with a diameter ranging from 30 to 40 nm. Larger particles are caused by the aggregation of single tiny particles that manage to fuse, producing a larger entity [61,62].

### 3.5. Energy-Dispersive Spectroscopy (EDS)

**Figure 7:**
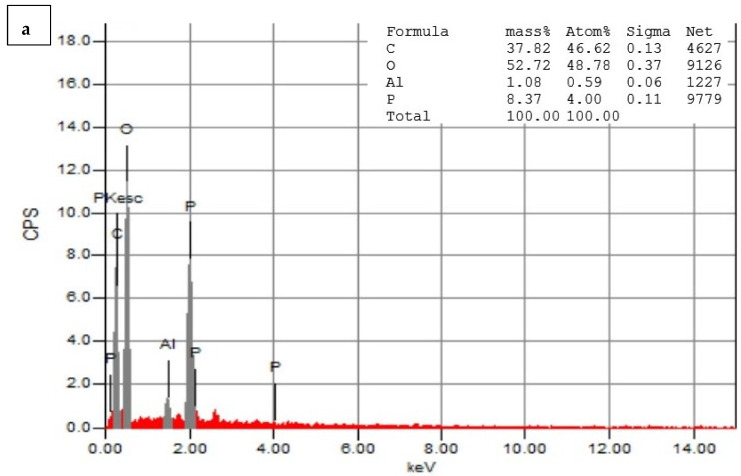

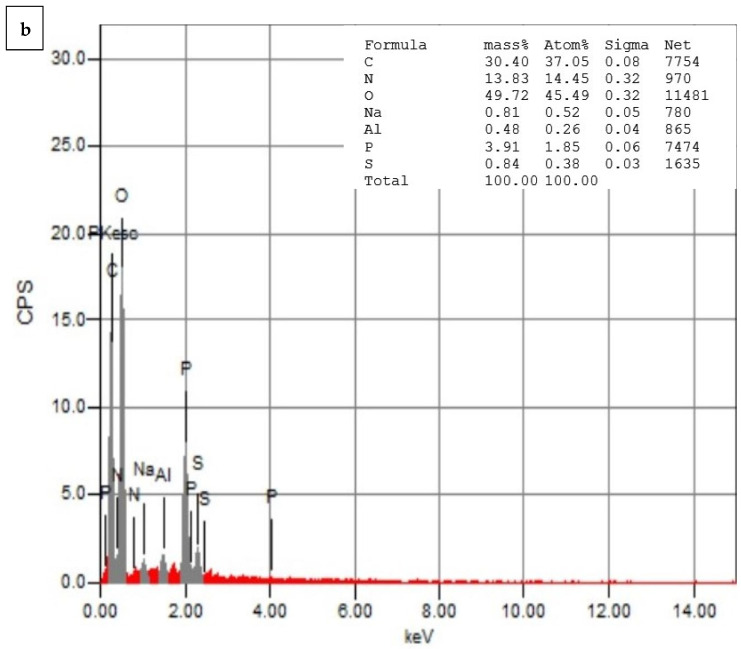
Energy-dispersive X-ray spectrophotometry analysis of CNPs (**a**) and CNPs/Cc (**b**).

Figure 7a,b depict the EDS analysis of the CNPs and the CNPs/Cc. The EDS of the CNPs revealed a high-intensity metallic peak of elements such as oxygen (O) with an atomic weight of 52.72%, 37.82% for carbon (C), 8.37% for phosphate (P), and low-intensity peaks of aluminum (Al). The EDS of the CNPs/Cc also confirmed the presence of carbon with an atomic weight of 30.40%, 49.72% for oxygen, 13.83% for nitrogen, 3.91% for phosphate, and low-intensity peaks of aluminum (Al), sodium (Na), and sulfur (S). It was possible to observe an increase in the intensities of the C and O signals relative to the other metals’ signals, which was likely caused by the chitosan nanoparticles [28]. EDS was employed to confirm the presence of TPP as cross-linked junctions in the CS particles, by monitoring the content of phosphorus in CS-TPP [63]. New signals of Na and P were obtained in the CNPs/Cc samples, due to the cross-linking of TPP with CS [64].

### 3.6. Differential Scanning Calorimetry (DSC)

**Figure 8:**
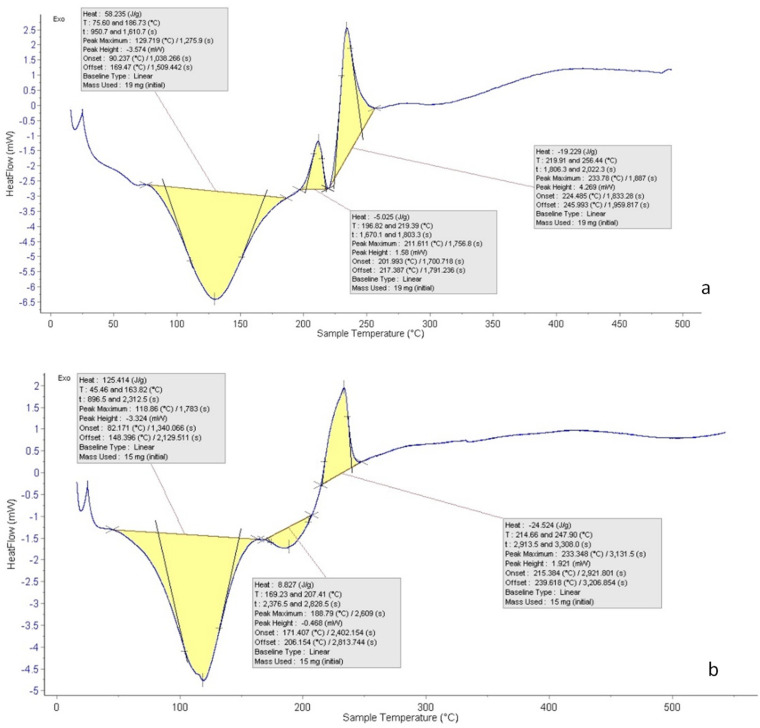
DSC of CNPs (**a**) and CNPs/Cc (**b**).

Figure 8 shows the DSC thermogram of the CNPs and the CNPs/Cc. The temperature ranged from 20 to 600 °C. A sharp endothermic peak was observed between 75.60 and 186.73 °C, showing the nanoparticles’ potential for heat breakdown. The exothermic peak of the CNP at 196.82 and 219.39.73 °C followed by an exothermic peak at 219.91 and 256.44 °C could be due to water evaporation. The CNPs exhibited a sharp endothermic peak at 45.46 and 163.82, after a tiny endothermic peak at 169.23 and 207.41, while an exothermic peak was observed at 214.66 and 247.90 °C. The result is in agreement with Yousef et al. [65], whose DSC thermogram of chitosan showed an endothermic peak at 180 °C and an exothermic peak at 370 °C. Chitosan DSC thermogram showed two peaks. The loss of water molecules was connected to the first one (endothermic, below 100 °C); the second signal, which was exothermic and occured at a temperature of about 300 °C, was connected to the breakdown of the chitosan pyranose ring [66,67]. The results showed that exothermic peaks, also known as crystallization peaks, were present throughout the DSC analyses’ heating scan [25].

### 3.7. Chromosomal Aberrations Observed in Bone Marrow Cells

Table 4 presents the number and percent of chromosomal aberrations in control and treatment animals. Treatment with EMS induced a significant increase in chromosomal aberrations in the bone marrow cells, reaching 24.4% (*p* < 0.01). The present results are in agreement with the findings of Norizadeh Tazehkand et al. [68], who documented that treatment of mice with 240 mg/kg EMS significantly increased chromosomal aberrations. There are large and diverse groups of chemicals that can induce DNA damage, and they are classified as carcinogens. Humans maybe exposed to these substances directly or indirectly in the environment or through diet. EMS has a potential negative effect, including cancer, aberrant birth outcomes, and heritable impacts, which are seen as natural consequences of its genotoxic activity [69]. EMS is an alkylating agent commonly used as a positive agent in genotoxicity studies [70] with the potential to cause cancer by altering DNA nucleotides, resulting in point mutations as the initial G:C base pair undergoes a mutation to A:T during replication base-pair insertions or deletions [71].

No significant aberrations were observed in the animal groups treated with CNPs or CNPs/Cc at their low tested doses. Moreover, pretreatment with CNPs/Cc at two tested doses for seven days succeeded in reducing (*p* < 0.01) the EMS-induced abnormalities significantly in a dose-dependent manner. The percentage of reduction reached 36.06% and 59.01% after treatment with low and high doses of CNPs/Cc, respectively (Table 4). Nanocurcumin and nanochitosan have antioxidant and antigenotoxic properties against the toxicity of potassium dichromate [72]. *Salix subserrata* bark extract-loaded chitosan nanoparticles exhibited promising potent neuroprotective and antioxidative efficiencies against arsenic-induced oxidative threats [73].

### 3.8. Sperm Shape Abnormalities

As shown in Table 5, male mice exposed to EMS developed defective sperm in a statistically highly significant (*p* < 0.01) percentage that reached 14.44%. The most prevalent anomalies were triangular, amorphous, and lacking hook heads and coiled tails. Chromosomal aberrations, testicular DNA alterations, and point mutations may contribute to the induction of sperm shape abnormalities [28].

The percentage of aberrant sperm was decreased when mice were treated with cCNP and CNPs simultaneously, reaching 10.02, 9.7, and 7.26% (*p* < 0.01). Following the treatment of mice with LD cCNP, HD cCNP and CNPs, the percentage of reduction reached 30.6, and 32.8, 49.7%. A previous study proved that pre-treatment with ZnO-Alg/NCMs for seven days significantly reduced chromosomal aberrations in somatic cells and spermatocytes and reduced the percentage of morphological sperm abnormalities [74].

Several studies on the phytochemistry of the genus *Capparis* have revealed the presence of terpenoids, flavonoids, alkaloids, glucosinolates, isothiocyanates, sterols, and fatty acids in various plant sections [9]. The methanol extract of *Capparis cartilaginea* leaves contains seven types of antioxidants [75] that could inhibit various human oxidative stress pathologies and possess DNA-protective attributes [76]. According to Moharram et al. [77], the anti-inflammatory activity is probably due to the antioxidant active constituents of the plant such as flavonoids, alkaloids, phenolic, coumarins, and tannin compounds.

Many recent studies emphasized the utilization of nanoparticles as a phytochemical carrier molecule to improve their solubility, bio-accessibility, and cellular bioavailability [78,79]. Several studies proved the protective capabilities of chitosan nanoparticles. Abdel-Wahhab et al. [80] demonstrated that chitosan nanoparticles modulate DNA fragmentation and suppress mycotoxin-induced genotoxicity in rats. The antioxidant and antigenotoxic properties of chitosan nanoparticles against potassium-dichromate-induced toxicity in male albino mice were documented by Mohamed et al. [72]. 

## 4. Conclusions

CNPs were synthesized, loaded with *C. cartilaginea* extract, and investigated via Fourier-transform infrared spectroscopy. The Zeta potential analyses proved that CNPs/Cc are more positively charged than CNPs. In vivo experiments illustrated that oral treatment with CNPs and CNPs/Cc is safe. Moreover, chitosan nanoparticles showed powerful antigenotoxic properties. Loading the chitosan nanoparticles with *C. cartilaginea* extract greatly improved their anticancer properties, mainly due to the plant’s active constituents and the small nanoparticle size that encouraged its cellular bioavailability. 

## Figures and Tables

**Table 1:** Experimental design and animal groups.

Groups	Treatment and Doses	Treatment Day(s)
Control	Control (negative)	1
EMS:	intraperitoneal single injection with 240 mg/kg b.w	
CNPs:	oral administration with 350 mg/kg b.w. of chitosan nanoparticle	
HD of CNPs/Cc:	oral administration with 700 mg/kg b.w. of CNPs loaded with *C. cartilaginea*	
LD of CNPs/Cc:	oral administration with 350 mg/kg b.w. of CNPs loaded with *C. cartilaginea*	
CNPs + EMS	CNPs (350 mg/kg b.w.) + EMS (single injection with 240 mg/kg b.w)	
HD of CNPs/Cc +EMS	(single injection with 240 mg/kg b.w)	
LD of CNPs/Cc + EMS	(single injection with 240 mg/kg b.w)	
Control	Control (negative)	7
EMS:	intraperitoneal single injection with 240 mg/kg b.w, 24 h before the experiment.	
CNPs:	oral administration with 350 mg/kg b.w. of chitosan nanoparticle	
HD of CNPs/Cc:	oral administration with 700 mg/kg b.w. of CNPs loaded with *C. cartilaginea*	
LD of CNPs/Cc:	oral administration with 350 mg/kg b.w. of CNPs loaded with *C. cartilaginea*	
CNPs + EMS	CNPs (350 mg/kg b.w.) + EMS (single injection with 240 mg/kg b.w)	
HD of CNPs/Cc +EMS	(single injection with 240 mg/kg b.w)	
LD of CNPs/Cc + EMS	(single injection with 240 mg/kg b.w)	

**Table 2:** *C. cartilaginea*, CNPs, and CNPs/Cc analysis by FTIR.

Wavenumber cm^−1^	Plant	CNP/Cc	CNPs	Functional Groups	Ref.
3346.88	Nd	D	Nd	Hydroxyl groups	[29]
3335.45	Nd	Nd	D	Stretching N-H asymmetric	[30]
3231.22	D	Nd	Nd	O-H bond stretching	[31]
2132.35	Nd	D	−6	Si–H stretching	[29]
1636.33	−38	D	D	CHO stretching of carbonyl group	[30]
1558.81	Nd	Nd	D	C–C stretch aromatic rings (phenolic)	[32]
1540.41	−22	D	D	Amide II	[30]
1418.53	−21	D	+39	Deformation C–H	[30]
1267.27	+	Nd	Nd	C-O stretching	[33]
1077.43	Nd	D	+19	C=C bond	[34]
1011.96	+17	D	Nd	C-F groups	[28]
950.72	Nd	D	Nd	Amines	[35]
614.09	D	Nd	+9	C–S stretch	[36]

D: detected, Nd: not detected, (−): shifted wavenumber by minus, (+): shifted wavenumber by addition.

**Table 3:** GC–MS analysis of *C*. *cartilaginea* extract.

Biological Activity	Molecular Weight	Molecular Formula	Chemical Structure	Area %	Compound Name	RT
Antimicrobial, Anti-inflammatory, Antioxidant [37]	152	C_9_H_12_O_2_	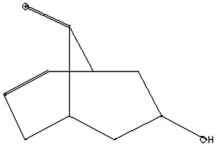	4.25	7-Hydroxy-bicyclo[3.3.1]n on-2-en-9-one	5.53
Antimicrobial, Anti-inflammatory, Antioxidant [37]	152	C_9_H_12_O_2_	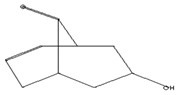	6.15	7-HYDROXY-BICYCLO[3.3.1]N ON-2-EN-9-ONE	5.58
Anticancer agent [38]	129	C_5_H_7_NO_3_	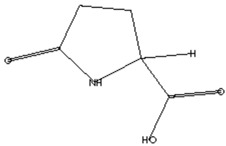	9.03	D-Pyroglutamic acid	6.50
Acetohydrazides and acetamides possessed anticancer agents [39]	196	C_10_H_16_N_2_O_2_	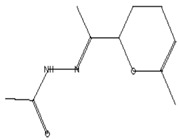	2.05	Acethydrazide, n2-[1-(2,3-dihydro-6-methyl pyran-2-yl)ethylideno]-	7.14
Analgesic, digestive, and wound healing [40] Agonist activity at human TRPA1 channel expressed in HEK293 cell [41]	150	C_10_H_14_O	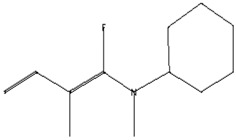	2.00	3,5-Heptadienal, 2-ethylidene-6-methyl-	13.36
The main compounds are found in Kei Apple fruits and have antioxidant and anticancer activities [42]	157	C_2_H_7_NO_3_S_2_	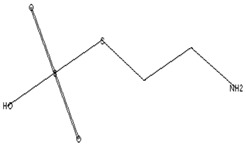	2.98	2-AMINOETHANETHIOL HYDROGEN SULFATE (ESTER)	24.73
Hepatoprotective Antiandrogenic, Antihistaminic, Anticoronary, Insectifuge, Anticancer [43]	298	C_18_H_31_ClO	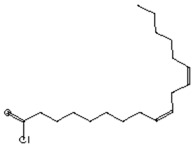	2.76	9,12-Octadecadienoyl chloride, (Z,Z)-	25.07
Anti-oxidant, decreases blood cholesterol, anti-inflammatory [44]	270	C_17_H_34_O_2_	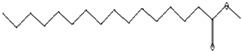	11.79	Hexadecanoic acid, methyl ester	26.13
Antimicrobial [45]	284	C_18_H_36_O_2_	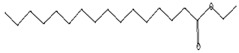	3.09	Hexadecanoic acid, ethyl ester	27.39
Antibacterial [46]	282	C_18_H_34_O_2_	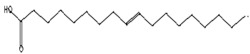	3.21	9-Octadecenoic acid (z)-, hexadecanoic acid	27.84
Antioxidant, anticancer [44,47]	296	C_19_H_36_O_2_	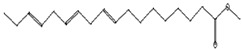	2.09	9-Octadecenoic acid (Z)-, methyl ester	29.31
Antimicrobial [48]	298	C_19_H_38_O_2_	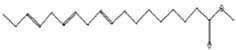	3.24	Octadecanoic acid, methyl ester	29.82
Antibacterial, Anti-inflammatory, cancer preventive, hepatoprotective, nematicide, insectifuge [45]	292	C_19_H_32_O_2_	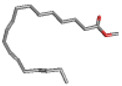	14.99	9,12,15-Octadecatrienoic acid, methyl ester	30.54
Antibacterial [46]	282	C_18_H_34_O_2_	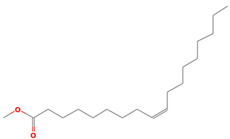	2.91	9-OCTADECENOIC ACID (Z)- 2-AMINOETHANETHIOL	30.96
Anti-atherosclerotic, anti-inflammatory, and anti-proliferative effects [49]	302	C_20_H_30_O_2_	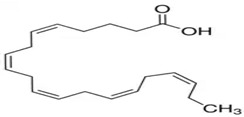 21,736,059.79	3.22	cis-5,8,11,14,17-Eicosapentaenoic acid	43.76

**Table 4:** Number and mean percentage of the different types of chromosomal aberrations in mouse bone marrow cells after treatment with different doses of CNPs/Cc and CNPs alone or in combination with EMS.

Treatment		No. of Metaphases with Aberrations					Chromosomal Aberrations		
	Gap	Frag. and/or Break	Gap + (Frag. or Break)	Deletion	Ring	Total No.	Excluding Gaps Mean ± S.E.	Including Gaps Mean ± S.E.	Inhibition %
One-day treatments									
Control	2	4	0	0	0	6	0.8 ± 0.4	1.2 ± 0.4	
EMS	20	62	28	10	2	122	20.4 ± 0.7 a	24.4 ± 0.4 a	
CNPs	10	9	5	0	0	24	3.6 ± 0.48	4.8 ± 0.4	
HD of CNPs/Cc	12	5	2	2	0	21	4.6 ± 0.2	4.2 ± 0.2	
LD of CNPs/Cc	13	3	6	1	0	23	4.2 ± 0.3	4.106 ± 0.4	
CNPs + EMS	14	50	33	11	0	108	18.8 ± 0.37	21.6 ± 0.7 a	11.4
HD of CNPs/Cc + EMS	11	61	15	9	1	97	17.2 ± 0.37	19.4 ± 0.6 a	20.49
LD of CNPs/Cc + EMS	22	51	33	0	0	106	16.8 ± 0.6 a	21.2 ± 0.6 a	13.1
Seven-day treatments									
Control	2	4	0	0	0	6	0.8 ± 0.4	1.2 ± 0.1	
EMS	20	62	28	10	2	122	20.4 ± 0.7 a	24.4 ± 0.4 a	
CNPs	12	3	5	2	0	23	2.2 ± 0.2	4.6 ± 0.6	
HD of CNPs/Cc	12	5	3	0	0	20	1.6 ± 0.4	4.0 ± 0.1	
LD of CNPs/Cc	12	10	0	0	0	22	2.0 ± 0.4	4.4 ± 0.4	
CNPs + EMS	20	39	11	12	0	82	12.4 ± 0.6 ab	16.4 ± 0.7 ab	32.7
HD of CNPs/Cc + EMS	12	27	4	7	0	50	7.6 ± 0.5 ab	10 ± 0.8 ab	59.01
LD of CNPs/Cc + EMS	18	24	32	4	0	78	12 ± 0.7 ab	15.6 ± 0.7 ab	36.06

a: Significant at 0.01 level (*t*-test) compared to control (non-treated). b: Significant at 0.01 level (*t*-test) compared to treatment (EMS).

**Table 5:** Number and percentage of different types of sperm shape abnormalities in male mice after treatment with different doses of CNPs/Cc and CNPs alone or in combination with EMS.

Treatment and Doses (mg/kg b.wt.)	Sperm No.	No. of Sperm with Abnormalities in							Abnormal Sperm No.	Abnormal Sperm Mean % ± S.E.	Inhibition %
		Head			Tail						
		Amorphous	Tringle	Without Hook		Small	Big	Coiled			
Control	5000	29	36	28		0	0	6	99	1.98 ± 0.1	
EMS	5000	259	183	47		3	1	229	722	14.4 ± 1.78 a	
CNPs	5000	31	25	6		0	0	24	86	1.72 ± 0.24	
HD of CNPs/Cc	5000	25	32	5		0	0	21	83	1.66 ± 0.2	
LD of CNPs/Cc	5000	32	21	7		0	0	35	95	1.9 ± 0.9	
CNPs + EMS	5000	204	109	19		2	0	167	501	10.02 ± 0.98 ab	30.6
HD of CNPs/Cc + EMS	5000	158	93	23		7	2	80	363	7.26 ± 0.56 ab	49.7
LD of CNPs/Cc + EMS	5000	106	130	30		4	1	214	485	9.7 ± 0.22 ab	32.8

a: Significant at 0.01 level (*t*-test) compared to control (non-treated). b: Significant at 0.01 level (*t*-test) compared to treatment (EMS).

## Data Availability

The datasets used and/or analyzed during this study are available from the corresponding author on reasonable request.
